# Production of Highly Active Recombinant Dermonecrotic Toxin of *Bordetella Pertussis*

**DOI:** 10.3390/toxins12090596

**Published:** 2020-09-15

**Authors:** Ondrej Stanek, Irena Linhartova, Jana Holubova, Ladislav Bumba, Zdenko Gardian, Anna Malandra, Barbora Bockova, Shihono Teruya, Yasuhiko Horiguchi, Radim Osicka, Peter Sebo

**Affiliations:** 1 Institute of Microbiology of the Czech Academy of Sciences, Videnska 1083, 142 20 Prague, Czech Republic; stanek@biomed.cas.cz (O.S.); linhart@biomed.cas.cz (I.L.); hejnova@biomed.cas.cz (J.H.); bumba@biomed.cas.cz (L.B.); anna.malandra@biomed.cas.cz (A.M.); bockova.ba@gmail.com (B.B.); 2 Institute of Parasitology, Biology Centre, The Czech Academy of Sciences, Branišovská 31, 370 05 České Budějovice, Czech Republic; gardian@prf.jcu.cz; 3 Department of Molecular Bacteriology, Research Institute for Microbial Diseases, Osaka University, Suita, Osaka 565-0871, Japan; teruya.s913@gmail.com (S.T.); horiguti@biken.osaka-u.ac.jp (Y.H.)

**Keywords:** Bordetella, GTPase, deamidation, dermonecrotic toxin, recombinant, electron microscopy, negative staining, image analysis

## Abstract

Pathogenic *Bordetella* bacteria release a neurotropic dermonecrotic toxin (DNT) that is endocytosed into animal cells and permanently activates the Rho family GTPases by polyamination or deamidation of the glutamine residues in their switch II regions (e.g., Gln63 of RhoA). DNT was found to enable high level colonization of the nasal cavity of pigs by *B. bronchiseptica* and the capacity of DNT to inhibit differentiation of nasal turbinate bone osteoblasts causes atrophic rhinitis in infected pigs. However, it remains unknown whether DNT plays any role also in virulence of the human pathogen *B. pertussis* and in pathogenesis of the whooping cough disease. We report a procedure for purification of large amounts of LPS-free recombinant DNT that exhibits a high biological activity on cells expressing the DNT receptors Cav3.1 and Cav3.2. Electron microscopy and single particle image analysis of negatively stained preparations revealed that the DNT molecule adopts a V-shaped structure with well-resolved protein domains. These results open the way to structure–function studies on DNT and its interactions with airway epithelial layers.

## 1. Introduction

*Bordetella pertussis* causes the respiratory infectious disease called pertussis, or whooping cough, which used to be the first cause of infant mortality prior to global introduction of efficient whole cell-based pertussis (wP) vaccines [1]. Still, whooping cough remains the least-controlled vaccine-preventable infectious disease and accounts for more than 24 million cases and 160,000 pertussis-linked deaths annually worldwide [2]. Moreover, two decades ago the wP vaccines were replaced in the most developed countries by the less reactogenic acellular pertussis (aP) vaccines that confer a less complete and shorter-lasting protection [3] that led to resurgence of pertussis outbreaks [4,5]. Therefore, development of improved pertussis vaccines, capable to control *B. pertussis* transmission in highly vaccinated populations, is required and calls for better understanding of the pathophysiology of *B. pertussis* infections. In particular, the virulence factors that enable the bacterium to colonize human nasopharynx need to be identified.

*B. pertussis* is a particularly well equipped pathogen that produces a number of secreted virulence factors. Among its several adhesive systems are the fimbriae (FIM), filamentous hemagglutinin (FHA), pertactin (PRN), and the tracheal colonization factor (TcfA). The Vag8 and BrkA autotransporter proteins confer complement resistance on the bacterium and the evasion of *B. pertussis* to bactericidal activities of phagocytes is mediated by the action of two potently immunosuppressive protein toxins, the adenylate cyclase toxin (CyaA) and the more notoriously known pertussis toxin (PT) [6]. Like other pathogenic bordetellae, *B. pertussis* also produces a heat-labile dermonecrotic toxin (DNT) that provokes formation of typical skin lesions upon intradermal injection [7,8,9]. While the role of DNT in *B. pertussis* infections remains unknown, the toxin was found to provoke lymphocyte depletion and reduced intensity of extramedullar monocytopoiesis in spleens of *B. bronchiseptica*-infected mice [10]. Moreover, DNT appears to be required for high level *B. bronchiseptica* colonization of porcine nasal cavity [11] and DNT activity provokes the atrophic rhinitis (AR) disease of pigs characterized by atrophy of the nasal turbinate bones [8,12]. This results from DNT-triggered inhibition of osteoblastic differentiation and bone formation, which provokes osteoclastic differentiation and bone resorption adjacent to degenerative osteoblasts and osteocytes [8,13,14,15,16,17].

DNT is a 1464 residue-long polypeptide [18] and its first 30 residues are crucial for toxin binding to the receptor on target cells [19,20]. A putative transmembrane domain, located between residues 45–166 of DNT was implicated in toxin translocation of and in delivery of a ~300 residue-long C-terminal transglutaminase enzyme domain into host cells [21,22,23]. The toxin enters target cells by dynamin-dependent endocytosis, undergoes cleavage between the Arg44 and Glu45 residues by a furin-like protease and translocates the C-terminal enzyme domain across the endosomal membrane into cell cytosol in an acidification-independent manner [23]. The transglutaminase enzyme then selectively deamidates or polyaminates the glutamine residues in the switch II region of Rho family GTPases (e.g., Gln63 of Rho, Gln61 of Rac, and Gln61 of Cdc42), ablating their GTP-hydrolyzing activity and locking them in the GTP-loaded state [17]. The irreversibly activated Rho GTPases then constitutively stimulate downstream signaling pathways and dysregulate various cellular processes. For example, cells intoxicated by DNT exhibit an aberrant organization of the actin cytoskeleton (including stress fibers, filopodia, and lamellipodia), multinucleation and stimulation of DNA, and protein synthesis [15,17,24,25,26]. Recently, the receptor molecules for DNT were identified as the Ca_v_3.1 and Ca_v_3.2 calcium channels encoded by the CACNA1G and CACNA1H genes [27].

Previously, several laborious methods for purification of native DNT from *Bordetella* cells were developed [9,28,29,30] and low yields of recombinant DNT from *E. coli* cells were achieved [19,22,23]. Therefore, we report a procedure for rapid purification of large amounts of LPS-free recombinant DNT and show that it is highly active and completely reproduces all known activities of native DNT.

## 2. Results

### 2.1. Cloning, Expression, and Purification of Recombinant DNT

**Figure 1:**
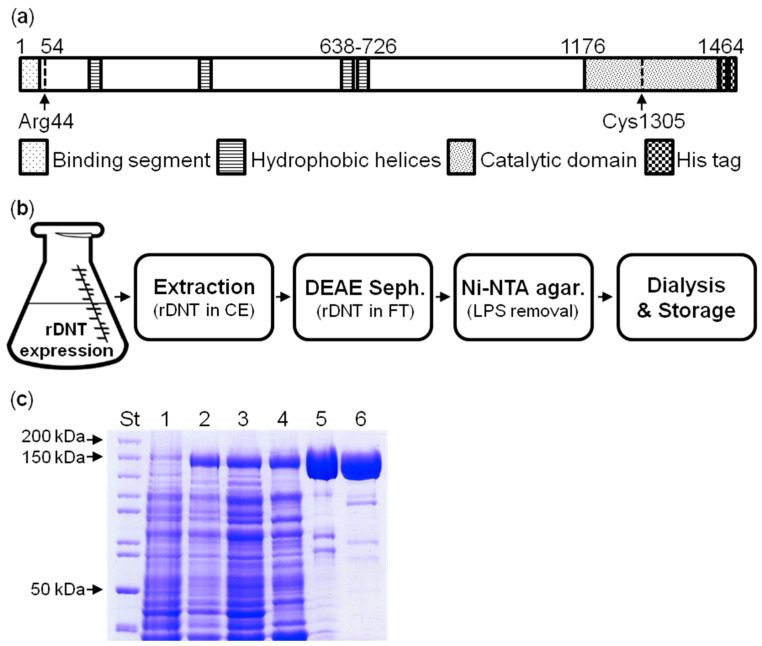
Purification of recombinant DNT. (**a**) Scheme of the recombinant rDNT molecule. The N-terminal segment (30 residues) mediates DNT binding to the cellular receptor (Cav3.1 or Cav3.2) and is followed by a furin-like cleavage site between residues Arg44 and Glu45. The central part of DNT contains four hydrophobic helices and the last 288 residues form the catalytic domain of a transglutaminase enzyme. Cys1305 is the catalytic residue replaced in the DNT-C1305S toxoid by a Ser residue. The rDNT molecule bears a C-terminal double-hexahistidine affinity purification tag. (**b**) Scheme of purification of rDNT. Clarified cytosolic extract of *E. coli* cells (CE) is passed over a DEAE Sepharose column in PBS at 7.4 to remove most of the contaminating proteins and the flow-through fraction containing rDNT is loaded onto Ni-NTA agarose. LPS and other contaminants are removed by excessive column wash with 10 bed volumes of 1% (*v*/*v*) Triton X-100 in PBS and detergent is removed by 5 bed volumes of PBS containing 100 mM imidazole. Bound rDNT is eluted with 500 mM imidazole (pH 8), dialyzed twice against 100 volumes of PBS and stored frozen in 50% glycerol at −80 °C (**c**) Coomassie-stained 7.5% SDS-PAGE of protein fractions. 1–uninduced *E. coli* cell lysate; 2–induced *E. coli* cell lysate; 3–cytosolic extract of induced *E. coli* cells; 4–DEAE Sepharose column flow-through fraction; 5–rDNT eluted from Ni-NTA agarose with 500 mM imidazole; 6–DNT in 50% glycerol after dialysis against PBS. ST, molecular weight standards.

To enable straightforward purification of the recombinant DNT protein, a sequence for double 6xHis purification tag was fused in frame to the 3’-end of the *dnt* open reading frame, inserted into the pET28b vector under the control of transcriptional and translational initiation signals of the gene 10 of bacteriophage T7 (Figure 1a).

High level production of soluble recombinant DNT protein was then achieved by expression of the *dnt* gene at 20 °C for 24 h in *Escherichia coli* Rosetta 2 cells that harbor tRNAs genes for rare codons and efficiently translate the mRNA transcripts of G:C-rich genes. The C-terminally tagged DNT protein was purified from the cytosolic fraction of bacterial lysates by a combination of pre-purification on DEAE Sepharose at pH 7.4 followed by ion metal affinity chromatography on Ni-NTA agarose (Figure 1b), where the contaminating *E. coli* outer membrane lipopolysaccharide (LPS) and other components were removed by extensive washing of the column with 1% (*v*/*v*) Triton X-100 [31]. The yield after the two purification steps was 3 mg of LPS-free (<100 EU/mg) DNT per 1 L of bacterial culture (Figure 1c). The same procedure was used to purify the catalytically inactive DNT-C1305S toxoid [22] used throughout this work as negative control.

### 2.2. Recombinant DNT Binds and Penetrates Target Cells, Deamidates Rho, and Triggers Remodeling of Actin Cytoskeleton

**Figure 2:**
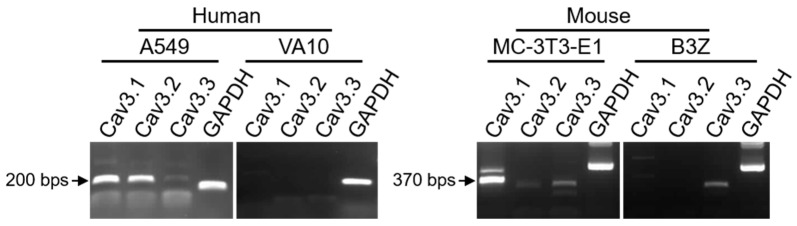
RT-PCR detection of the Cav3.1, Cav3.2, and Cav3.3 gene expression in different cell lines. Total RNA extracted from the indicated cells was reverse transcribed and the expression of individual calcium channel genes was detected by amplification of corresponding cDNA fragments with specific primer pairs (Table 1).

In order to compare the biological activities of the purified rDNT protein on cells that produce or lack the recently identified Cav3.1 and Cav3.2 receptors of DNT [27], we first screened a set of cell lines for expression of the T-type calcium channels (Cav3.1, Cav3.2, and Cav3.3). As shown in Figure 2, RT-PCR detection with gene-specific primers (Table 1) revealed that human lung epithelial A549 cells express both calcium channels Cav3.1 and Cav3.2, whereas the mouse preosteoblast MC-3T3-E1 cells only expressed higher levels of Cav3.1. No, or very low level of expression of the T-type calcium channels was then detected in the differentiated human bronchial epithelial VA10 cells and in the mouse T-cell hybridoma B3Z cells (Figure 2).

**Figure 3:**
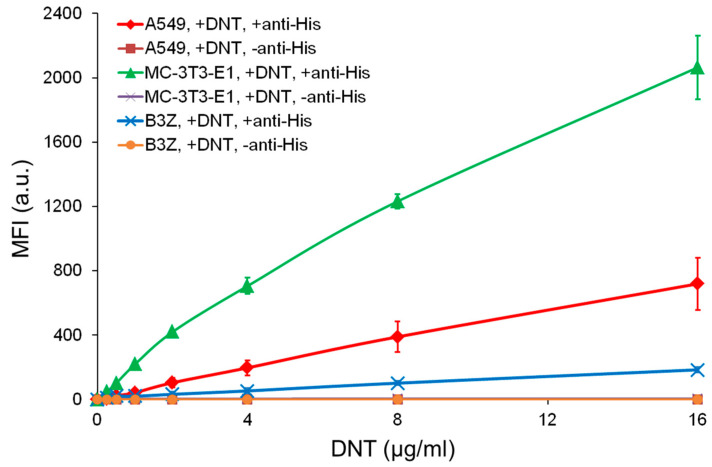
DNT binding to three types of mouse and human cells. 2 × 10^5^ of human A549, or mouse MC-3T3-E1 and B3Z cells were incubated for 30 min at 4 °C with different concentrations of rDNT bearing a C-terminal double 6xHis tag, the surface-bound toxin was labeled with an anti-6xHis tag antibody (omitted in negative control samples) and decorated by a secondary antibody conjugated with Alexa Fluor 488. The cells were analyzed by flow cytometry and mean fluorescence intensities of DNT binding were plotted against the concentrations of DNT. Each point represents the mean value ± SD of three independent experiments. MFI, mean fluorescence intensity.

In line with that, the rDNT bound MC-3T3-E1 and A549 cells in a dose-dependent manner, while only low binding of DNT to B3Z cells was observed (Figure 3). The human A549 cells expressing both Cav3.1 and Cav3.2 bound DNT with ~4-fold lower efficacy (calculated for DNT concentrations ranging from 0.5 to 16 µg/mL) than mouse MC-3T3-E1 cells expressing only theCav3.1.

**Figure 4:**
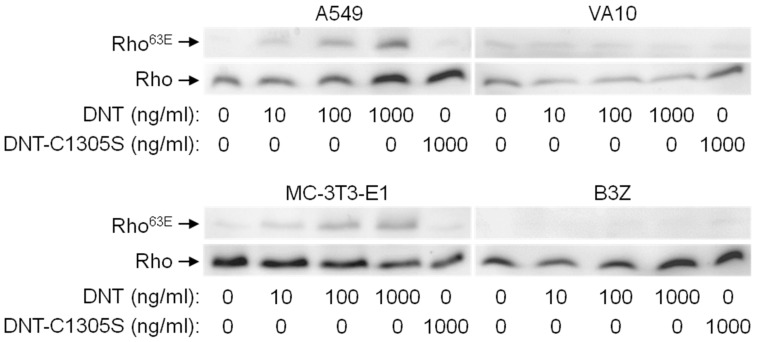
DNT, but not DNT-C1305S deamidates the Gln63 residue of RhoA in A549 and MC-3T3-E1 cells. Four different cell lines (A549 and VA10–human; MC-3T3-E1 and B3Z–mice) were treated with different concentrations of DNT (0, 10, 100, and 1000 ng/mL) or with 1000 ng/mL of the DNT-C1305S mutant for 4 h. Then the cells were lysed, and proteins were separated by Tris-Tricine SDS-PAGE. The separated proteins were transferred onto a nitrocellulose membrane, where the deamidation of the Gln63 residue of RhoA was detected by an anti-Rho^63E^ antibody and the total RhoA was determined by an anti-RhoA antibody.

We next analyzed whether the purified rDNT toxin was able to penetrate cells and specifically deamidate Gln63 of RhoA to produce RhoA^63E^ that is detectable by a specific antibody. As shown in Figure 4, the rDNT protein was highly active, since deamidated RhoA^63E^ was detectable in human A549 and mouse MC-3T3-E1 cells exposed to as little as 10 ng/mL of rDNT for 4 h and the RhoA^63E^ amounts increased with increasing concentrations of rDNT. In contrast, no RhoA^63E^ formation was detected upon treatment with the enzymatically inactive rDNT-C1305S toxoid, serving as a negative control. The cell penetration activity of rDNT depended on the interaction with cellular receptor, as no formation of RhoA^63E^ was observed in VA10 and B3Z cells even upon exposure to 1000 ng/mL of rDNT.

**Figure 5:**
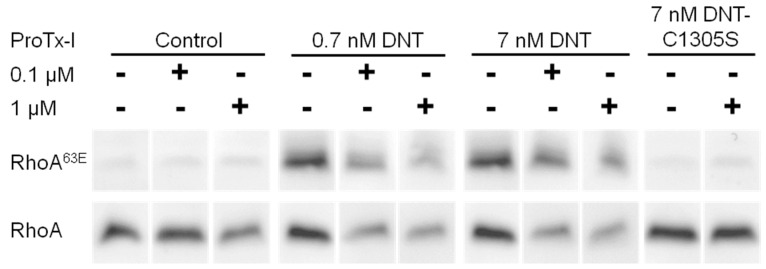
ProTx-I blocks the entry of DNT into cells. MC-3T3-E1 cells were pretreated with different concentrations of ProTx-I (0, 0.1, and 1 µM) for 1 h. Then the cells were incubated with DNT or DNT-C1305S for next 4 h, lysed, and the deamidation of RhoA was determined on Western blots as described in the legend to Figure 4. The used 0.7 nM DNT corresponded to 100 ng DNT/mL.

Indeed, rDNT penetration and RhoA^63E^ formation in the sensitive MC-3T3-E1 cells could be inhibited in part in the presence of excess of the ProTx-1 peptide (Figure 5) that binds the Cav3.1 receptor [27].

**Figure 6:**
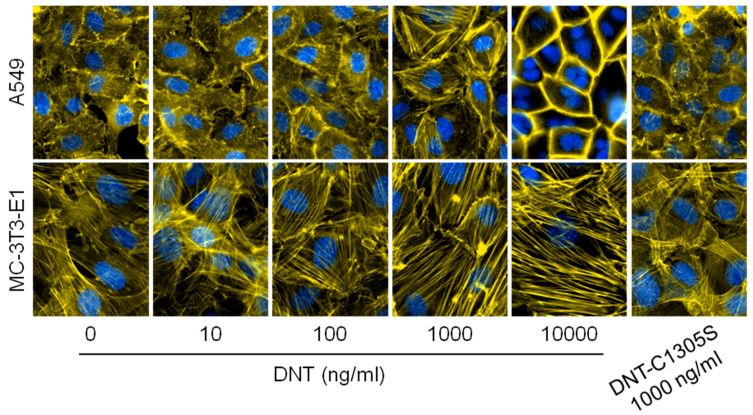
rDNT, but not rDNT-C1305S induced formation of stress fibers and/or alteration of cortical actin cytoskeleton. Human lung epithelial cells A549 and mouse preosteoblast cells MC-3T3-E1 were exposed to increasing concentrations of rDNT, or to 1000 ng/mL of the DNT-C1305S toxoid for 12 h. The cells were fixed with 4% paraformaldehyde, stained for F-actin with phalloidin-TRITC and for nuclei with DAPI and examined using an Olympus microscope IX81.

As shown in Figure 6, the capacity of rDNT to penetrate cells and activate Rho GTPases translated into remodeling of actin cytoskeleton of the A549 and MC-3T3-E1 cells exposed to rDNT for 12 h. Increased formation of actin stress fibers was observed in the more sensitive MC-3T3-E1 cells already upon exposure to 10 ng/mL of rDNT. In contrast, rDNT action on A549 cells rather triggered formation of cortical actin cytoskeleton and at high at rDNT concentrations (10 µg/mL) multinucleated cells were observed. These changes were elicited by the enzymatic activity of DNT on Rho GTPases, since even high concentrations (1000 ng/mL) of the catalytically inactive DNT-C1305S toxoid did not elicit any cytoskeletal rearrangements in the A549 and MC-3T3-E1 cells.

It remains unclear if the capacity of DNT to remodel actin cytoskeleton of cells might have any impact on the integrity of the barrier function of airway epithelia colonized by *Bordetella* bacteria. To address this question, we could only use the model of polarized pseudostratified human layers made of VA10 cells that do not express the DNT receptor. As a result, even high concentrations of rDNT (1 µg/mL) added form the apical side had no impact on the trans-epithelial electrical resistance (TEER) across the VA10 layers (data not shown).

### 2.3. Recombinant DNT Has Strong Dermonecrotic Effect in an Infant Mouse Model

**Figure 7:**
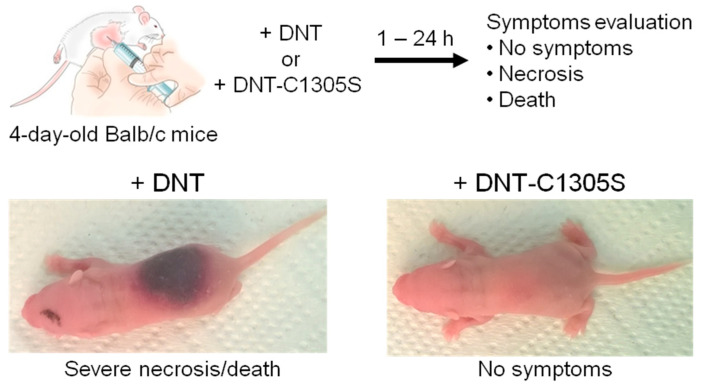
Dermonecrotic toxin assay. The suckling 4-day-old Balb/c mice were inoculated s.c. into nuchal area with 1 µg DNT or DNT-C1305S. Symptoms like severe necrosis and death were monitored over 24 h. Representative figures of DNT and DNT-C1305S inoculated animals are shown.

To corroborate the biological activity of the purified LPS-free rDNT protein, we assessed its dermonecrotizing activity by inoculating 1 µg of the toxin s.c. into nuchal area of suckling 4-day-old Balb/c mice. As shown in Figure 7, all six animals inoculated with DNT developed large necrotic areas surrounded by an inflammatory zone and four of the mice died within 24 h from inoculation. In contrast, all animals inoculated with DNT-C1305S toxoid remained without any lesions or pathology signs (Figure 7). These results demonstrate that the highly purified and LPS-free rDNT toxin possessed the high cytotoxic activity of the native *B. pertussis* DNT and can be used in structure–function studies and for analysis of its role in *B. pertussis* DNT activities in tissue models.

### 2.4. DNT is a Monomeric Protein with V-Shaped Structure Consiting of Two Extended Arms Linked by a Central Segment

**Figure 8:**
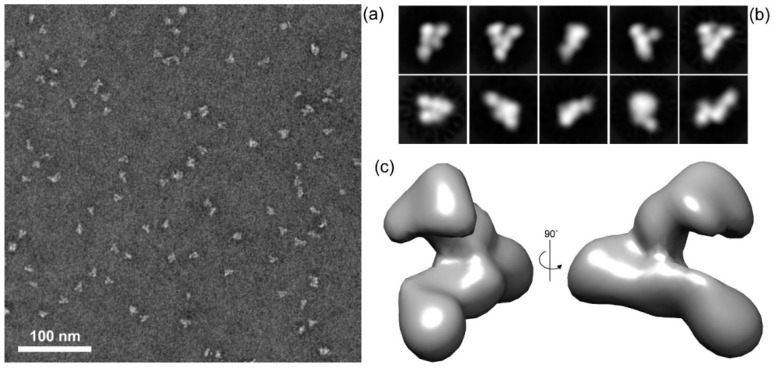
Structural analysis if DNT by single particle image analysis. (**a**) Representative TEM micrograph of negatively stained rDNT particles. (**b**) Averaged rDNT structures obtained by reference-free 2D classification of rDNT particles (left to right-top to bottom panels represent 1284, 1046, 953, 865, 853, 745, 727, 724, 722, 446 particles, respectively). (**c**) Final reconstructed 3D volume representation of rDNT particles.

To obtain some insight into DNT structure, we used negative staining transmission electron microscopy (TEM) with single particle image analysis. A homogenous preparation of the LPS-free rDNT was obtained by size exclusion chromatography on a Superdex 200 column form which rDNT eluted as a single peak with retention time corresponding to about 150 kDa, indicating that rDNT was monomeric. The protein was deposited on glow-discharged carbon-coated copper grids, negatively stained with uranyl acetate and visualized by TEM. The micrographs revealed that the preparation consisted of highly homogenous rDNT particles, and very few aggregates or degradation products were present (Figure 8a). A total of 22,404 particles were extracted from 29 micrographs and subjected to the reference-free two-dimensional (2D) classification to remove the noisy particles. The 2D classification resulted in 12 well-defined classes of 8365 particles that were selected for further processing.

The most representative 2D class averages of the selected dataset are depicted in Figure 8b. Nine out of 10 class averages revealed distinct views of a novel type of V-shaped particle with well-resolved structural features. The last class average showed an elongated protein structure that most likely represents a distinct rDNT conformer. To determine the three-dimensional (3D) structure of rDNT, the particles belonging to nine 2D classes were selected and subjected to 3D classification and refinement. This process yielded the final 3D density map of rDNT at 27 Å resolution, based on the gold-standard Fourier shell correlation (FSC) criterion calculated from two independent halves of the dataset (Figure 8c). The unusual DNT structure is composed of two comparably long and extended ‘arms’ that are linked by a central segment presumably comprising the membrane translocation domain of DNT. Since the globular domains at the ends of the ‘arms’ appeared to have comparable shape and size it was not possible to unambiguously fit into either one of them the homologous model for the catalytic domain of DNT built on the structure of the homologous CNF1 protein structure [32]. These data, however, show that obtaining higher resolution structures with the here reported quality of biologically active rDNT preparations and at varying pH and in detergents can be attempted.

## 3. Discussion

The involvement of the heat-labile dermonecrotic toxin in virulence of *Bordetella pertussis* and in pathogenesis of the whooping cough disease remains enigmatic. The high degree of sequence conservation of the *dnt* gene in *Bordetella* species pathogenic to birds and mammals and the expression of the *dnt* gene under the control of BvgAS, the master regulator *Bordetella* virulence genes would suggest that DNT might play a role in colonization of human nasopharynx by *B. pertussis*. Indeed, production of functional DNT appears to be required for a full capacity of *B. bronchiseptica* to persistently colonize the nasal epithelium of pig snout [11]. However, *B. pertussis* is an exclusively human pathogen and a subtle phenotype of a *dnt* mutant would likely not be revealed in the widely used mouse model of transient lung colonization by *B. pertussis*, which does not adequately reproduce the pathophysiology of human nasopharynx infection. Therefore, the role of DNT in the capacity of *B. pertussis* to colonize the nasal cavity of mice and primates needs to be addressed [33,34].

Intriguingly, DNT is only released by the bacteria upon lysis and Teruya and co-workers (2020) recently proposed that release of the neurotropic DNT upon treatment of the pertussis patients with beta lactam antibiotics might account for the cases of pertussis encephalopathy. In the absence of antibiotic treatment, some DNT amounts could be released onto host airway mucosa by spontaneous lysis of the bacteria, following permeabilization of *B. pertussis* cells by host antimicrobial peptides, upon killing by the membrane attack complex of complement, or inside phagocytic cells.

The here-reported procedure for straightforward production and purification of high amounts of fully biologically active and LPS-free recombinant DNT protein enabled a preliminary characterization of DNT structure by negative staining transmission electron microscopy with single particle image analysis (*c.f.* Figure 8). This revealed a completely novel and unusual extended V-shaped structure of the toxin molecule with two extended arms linked by a central segment that likely corresponds to the membrane translocation domain. The catalytic domain could not be unambiguously localized in the reconstructed volume structure and alternative approaches to fitting of its homologous model into the shown structure are currently explored. The homogeneity and stability of the here reported rDNT preparations now opens the way to further structural analysis at higher resolution, such as by X-ray crystallography of intact and mutated/tagged DNT variants alone, or in complex with specific monoclonal antibody Fab fragments, or at endosomal pH and in the presence of membrane/mimicking detergents. Easy preparation of specific DNT variants will facilitate the elucidation of the structure–function relationships underlying DNT translocation across the endosomal membrane into target cell cytosol. Finally, straightforward fluorescent labelling and recognition of the double 6xHis tag will enable tracking and visualization of the DNT protein in polarized epithelial layers and epithelial organoid models *in vitro* and tracking of the labelled DNT *in vivo* by intravital fluorescence microcopy and in histological sections of mouse nasal tissue. The availability of sufficient amounts of purified recombinant DNT protein and of its DNT-C1305S toxoid will likely enable the identification of target cells to which DNT binds in nasal epithelia. It may shed some light on whether DNT plays a role in *B. pertussis* colonization of airway mucosa and whether it can be trafficked through axons of olfactory neurons into the brain to cause pertussis encephalopathy, as recently proposed [27].

## 4. Materials and Methods

### 4.1. Bacterial Strains and Growth Conditions

The *E. coli* K12 strain XL1-Blue (Stratagene, La Jolla, CA, USA) was used throughout this work for DNA manipulation and was grown in Luria-Bertani (LB) medium supplemented with 60 µg/mL kanamycin. The *E. coli* strain Rosetta 2 (Novagen, Madison, WI, USA) was used for expression of recombinant proteins and was grown at 30 °C in LB medium containing 60 µg/mL kanamycin.

### 4.2. Plasmid Constructs

To construct the plasmid for expression of recombinant DNT, the *dnt* gene was amplified from the genomic DNA of the *B. pertussis* strain 1917 and the PCR product was cloned as an Nco I-Hind III fragment into the multiple cloning site of a pET28b-derived vector. In addition, the gene for DNT was fused in frame at the 3′-end to a sequence encoding a double-hexahistidine purification tag (6 × poly-His-loop-6 × poly-His) [35]. The resulting construct pET28b-*Bp*DNT was used for expression of the recombinant DNT protein in *E. coli* cells. Oligonucleotide-directed PCR mutagenesis was performed to construct pET28b-*Bp*DNT-C1305S plasmid for the expression of DNT with a point substitution of a cysteine residue with a serine residue at position 1305 (DNT-C1305S). All constructed plasmids were systematically verified by DNA sequencing.

### 4.3. Expression and Purification of Recombinant DNT

The DNT toxin and its DNT-C1305S variant were produced in *E. coli* Rosetta 2 cells in 1000 mL cultures in Luria-Bertani medium containing 60 µg/mL kanamycin. Cultures were grown with shaking at 28 °C to an optical density at 600 nm of 0.6, when the toxin expression was induced by adding of IPTG to a final concentration of 0.1 mM. The cultivation temperature was decreased to 20 °C and the cultures were grown for additional 20 h. Then the cells were harvested by centrifugation, resuspended in 40 mL of ice-cold phosphate buffered saline (PBS) pH 7.4, and disrupted by sonication at 4 °C. The homogenate was centrifuged at 20,000× *g* for 30 min at 4 °C and the clarified cytosolic extract was loaded onto a column with 10 mL of packed DEAE Sepharose CL-6B resin (Sigma–Aldrich, St. Louis, MO, USA) equilibrated with PBS. While most of *E. coli* contaminants were bound to the beads, DNT passed through the column. The pooled fractions containing the toxin were supplemented with NaCl to a final concentration of 300 mM and loaded on 5 mL Ni-NTA agarose column (GE Healthcare BioSciences, Pittsburgh, PA, USA) equilibrated with PBS containing 300 mM NaCl (equilibration buffer). To remove contaminating *E. coli* outer membrane lipopolysaccharide (LPS) and other components, the column was extensively washed with 10 bed volumes of equilibration buffer containing 1% (*v*/*v*) Triton X-100 (Sigma–Aldrich, St. Louis, MO, USA). Detergent was next removed by 5 bed volume washes with equilibration buffer containing 100 mM imidazole. Finally, DNT was eluted with PBS containing 500 mM imidazole, dialyzed two-times against 100 volumes of PBS to remove imidazole and stored in 50% glycerol at −80 °C (long term storage) or at −20 °C (short term storage). All purification steps and dialysis were done at 4 °C to prevent the degradation of DNT.

For structural analysis by electron microscopy 1 mg of rDNT was further purified by size exclusion chromatography on a Superdex 200 column (GE Healthcare) equilibrated in PBS buffer pH 7.4 using an AKTA Prime Plus instrument at a flow rate of 0.5 mL/min. The monomeric rDNT eluted as a single peak at a retention time corresponding approximately to a protein of 150 kDa.

### 4.4. Determination of LPS Levels and Protein Concentrations

The LPS levels in DNT samples were determined by the Chromogenic Limulus Amebocyte Lysate (LAL) endotoxin assay (Charles River Endosave, Charleston, SC, USA) and protein concentration was determined by the Bradford assay using BSA as the calibration standard. The purified protein was considered LPS-free at <100 endotoxin units (EU) per 1 mg of recombinant DNT, which was the detection limit of the LAL assay at the used DNT dilutions.

### 4.5. Cell Lines

Human alveolar basal epithelial cells A549 (ATCC, number CCL-185) and mouse T-cell hybridoma B3Z cells [36] were cultured in Dulbecco’s Modified Eagles Medium (DMEM; Thermo Fisher Scientific, Waltham, MA, USA), and MC3T3-E1 cells (ATCC, number CRL-2593) were cultivated in Minimum Essential Medium α (Thermo Fisher Scientific, Waltham, MA, USA). Both media were supplemented with 10% (*v*/*v*) fetal calf serum (FCS; GIBCO Invitrogen, Grand Island, NY, USA) and antibiotic antimycotic solution (0.1 mg/mL streptomycin, 100 U/mL penicillin and 0.25 mg/mL amphotericin; Sigma–Aldrich, St. Louis, MO, USA). An E6/E7 viral oncogene immortalized human bronchial epithelial cell line VA10 was maintained in bronchial/tracheal epithelial cell growth medium (B/TEGM; Cell applications, San Diego, CA, USA) with antibiotic-antimycotic solution. All cells were cultured at 37 °C in a humidified air/CO2 (19:1) atmosphere.

### 4.6. Air-Liquid Interface (ALI) Cultures of VA10 Cells

ALI cultures were set up on 0.4 µm transwell permeable filter supports (Corning Costar Corporation, Tewksbury, MA, USA), where VA10 cells were seeded in LHC9 growth medium (Thermo Fisher Scientific, Waltham, MA, USA) without addition of supplementary nutrients or antibiotics. Two to four days later, the medium was changed to DMEM:Nutrient mixture F-12 (Thermo Fisher Scientific, Waltham, MA, USA) supplemented with 2% serum substitute Ultroser G (PALL Life Sciences, Port Washington, NY, USA) and antibiotic-antimycotic solution on both apical and basolateral sides. Two to four days later, the medium was removed from the apical surface and the cells were cultured at the ALI for 14 to 21 days, with media changed every second day. Transepithelial electrical resistance (TEER) of VA10 cell layers was measured with a Millicell-ERS volt-ohm meter (Millipore, Burlington, MA, USA) and only the layers that generated TEER of at least 350 Ω.cm^2^, after subtraction of background resistance of empty transwell filters, were used for further studies.

### 4.7. Analysis of the Expression of Genes Encoding Calcium Channels Cav3.1, Cav3.2 and Cav3.3 in Different Cell Types

5 × 10^5^ cells (A549, MC-3T3-E1, B3Z and polarized VA10) were lysed by 0.5 mL of TRI Reagent solution (Sigma–Aldrich, St. Louis, MO, USA) and total RNA was extracted using an RNeasy Mini Kit (Qiagen, Hilden, Germany). The integrity of the isolated RNA was verified by electrophoresis on agarose gel and the purity and concentration of RNA were determined using NanoDrop (Thermo Scientific, Waltham, MA, USA). A total of 1 μg of the isolated total RNA was used as template for reverse transcription into cDNA in a 20 μL reaction mixture using a QuantiTect Reverse Transcription Kit (Qiagen, Hilden, Germany) following the manufacturer’s instructions. Next, 1 μg of total cDNA and 200 nmol/L of each primer (Table 1) were used together with the Q5 High-Fidelity DNA Polymerase (NEB, Ipswich, MA, USA) in a 20 µL PCR reaction volume to amplify genes encoding calcium channels Cav3.1, Cav3.2, and Cav3.3. PCR amplification was performed using the T100 Thermal Cycler (Bio-Rad, Hercules, CA, USA) with the initial step at 98 °C for 3 min, followed by 35 cycles at 98 °C for 20 s, 58 °C for 30 s, and 72 °C for 40 s. The PCR products were analyzed on a 2% agarose gel stained with ethidium bromide and visualized on a G:BOX (Syngene, Cambridge, UK).

### 4.8. Determination of DNT Binding to Target Cells

Cultivated cells were harvested and washed in HEPES-buffered salt solution (10 mM HEPES (pH 7.4), 140 mM NaCl, 5 mM KCl) supplemented with 2 mM CaCl_2_, 2 mM MgCl_2_, 1% (*w*/*v*) glucose and 1% (*v*/*v*) FCS (cHBSS buffer). We incubated 2 × 10^5^ cells in 100 µL of cHBSS buffer with different concentrations of purified DNT for 30 min at 4 °C. Unbound toxin was washed out by rinsing with cHBSS buffer and cells with the surface-bound DNT were incubated in 100 µL of cHBSS buffer with mouse anti-His mAb (diluted 1:1000; Sigma–Aldrich, St. Louis, MO, USA) recognizing the C-terminal double His tag of the toxin for 30 min at 4 °C. Cells were washed with cHBSS buffer and incubated in 100 µL of cHBSS buffer with AlexaFluor488-conjugated F(ab’)_2_ fragment goat anti-mouse IgG antibody (diluted 1:400; Jackson ImmunoResearch Europe Ltd., Cambridgeshire, UK) for 30 min at 4 °C. After washing, cells were resuspended in 100 µL of cHBSS and analyzed by flow cytometry on a FACS LSR II instrument (BD Biosciences, San Jose, CA) in the presence of 1 µg/mL of Hoechst 33258. Data were analyzed using the FlowJo software (Tree Star, Ashland, OR) and appropriate gatings were used to exclude cell aggregates and dead cells. The negative control for background anti-His antibody binding without DNT but in the presence of both anti-His and secondary antibody is shown in Figure 3 as the mean fluorescence intensity (MFI) at 0 μg/mL of DNT + anti-His. This background value was the same as in the absence of the anti-His secondary antibody, clearly showing that the binding of anti-His was highly specific and was fully dependent on the amount of cell-bound DNT in a concentration dependent manner.

### 4.9. Immunofluorescent Staining of A549 and MC-3T3-E1 Cells and Fluorescent Microscopy

A549 and MC-3T3-E1 cells were seeded on coverslips (Ø 12 mm) at a density of 2 × 10^4^ cells per cm^2^. After 24 h incubation at 37 °C and 5% CO_2_ in complete medium (DMEM with 10% FCS), the cells were treated 12 h with various concentrations of DNT or DNT-C1305S in complete medium. Then the cells were washed with PBS, fixed in 4% PFA for 20 min, permeabilized by 0.2% Triton X-100 for 10 min and blocked by 1 h incubation in 5% BSA. For staining, the cells were incubated for 45 min with 20 µg/mL of phalloidin/TRITC (Sigma–Aldrich, St. Louis, MO, USA) and 4 µg/mL of DAPI (Sigma–Aldrich, St. Louis, MO, USA), both diluted in PBS buffer supplemented with 2% BSA and 0.02% Triton X-100. After rinsing with the same buffer, the coverslips were mounted in Vectashield mounting medium (Vector Laboratories, Burlingame, CA, USA) and observed on an Olympus CellR system (IX81 microscope at 60× magnification).

### 4.10. Western Blot Analysis of Deamidation of Gln63 of RhoA

We treated 5 × 10^5^ A549, MC-3T3-E1, B3Z, and polarized VA10 cells with 10, 100, or 1000 ng/mL of endotoxin-free recombinant DNT or its DNT-C1305S variant used as negative control. After 4 h of incubation at 37 °C, the cells were lysed with 50 mM Tris-HCl (pH 8.0), 200 mM DTT, 0.3% SDS, Complete mini inhibitors (1 tablet per 10 mL, Merck, Darmstadt, Germany), heated for 3 min at 99 °C and then treated with benzonase (Novagen, Madison, WI, USA) for 30 min at 4 °C. The lysates containing 10 µg of the total protein were separated by Tris-Tricine SDS-PAGE and transferred to nitrocellulose membranes. The deamidated RhoA bearing Glu63 was detected by the anti-RhoA^63E^ antibody 6MmDNT22–2 at a 1:250 dilution (provided by Yasuhiko Horiguchi, Osaka University), while total RhoA was detected by the anti-RhoA antibody EP487Y at a 1:1000 dilution (Abcam, Cambridge, UK). Primary antibodies were then probed by peroxidase-conjugated anti-rabbit secondary antibody at a 1:5000 dilution (GE Healthcare BioSciences, Pittsburgh, PA, USA). After washing, the membranes were incubated with a SuperSignal Sensitivity Substrate kit (Thermo Fisher Scientific, Waltham, MA, USA) and the chemiluminescence signal was detected using a G:BOX Chemi XRQ imaging system (Syngene, Cambridge, UK).

### 4.11. Blocking of Recombinant DNT Entry into Cells by ProTx-I

We pretreated 5 × 10^5^ MC-3T3-E1 cells with 0.1 or 1 µM ProTx-I (Tarantula *Thrixopelma pruriens* venom, Peptide Institute Inc., Osaka, Japan) for 1 h at 37 °C and without a wash, DNT or DNT-C1305S ptoteins were added to 0.7 or 7 nM final concentration and the incubation was continued for 4 h at 37 °C. The cells were next lysed and the total and deamidated forms of RhoA were detected by Western blots.

### 4.12. Dermonecrotic Activity of Recombinant DNT on Mice

Four-day-old suckling female mice Balb/c, obtained from a breeding colony at the Institute of Molecular Genetics of the Czech Academy of Sciences in Prague, Czech Republic, were inoculated subcutaneously in the nuchal area with 1 µg of endotoxin-free recombinant DNT or its DNT-C1305S variant and the symptoms like necrosis or dead were observed few hours after the injection. This assay is a standard control used in the whole cell pertussis vaccine testing procedure (WHO Manual for Quality Control of Diphtheria, Tetanus and Pertussis Vaccines, 2013).

All animal experiments were approved by the Animal Welfare Committee of the Institute of Molecular Genetics of the Czech Academy of Sciences under the permission number 89/2019 (approved date: 29 October 2019). Handling of animals was performed in accordance with the Guidelines for the Care and Use of Laboratory Animals, the Act of the Czech National Assembly, Collection of Laws No. 246/1992.

### 4.13. Structural Analysis of rDNT Particles by Single Particle Image Analysis

rDNT protein freshly eluted from the Superdex 200 column was diluted to 10 µg/mL in PBS pH 7.4, applied to glow-discharged carbon-coated copper grids and allowed to adhere for 10 s before being reduced to a thin film by blotting. Immediately after, 3 μL of a 2% solution of unbuffered uranyl acetate was applied to the grid and blotted-off directly. The grids desiccated at room temperature were imaged using a Jeol JEM-1400 transmission electron microscope operated at 120 kV and equipped with a bottom-mounted FLASH 2k × 2k CMOS camera. Image processing was performed using XMIPP3 [40] and Relion 3.0 [41].

## Figures and Tables

**Table 1:** List of primers used to amplify genes encoding calcium channels Cav3.1, Cav3.2, and Cav3.3.

Gene ^1^	Oligonucleotide Sequence (5′→3′)	Amplification Size (bp)	Accession Number	Ref.
**h*Cacna1g***	F: GCTCTTTGGAGACCTGGAGTGT	197	NM_198382	[37]
	R: TAGGCGAGATGACCGTGTTG			
**h*Cacna1h***	F: TTGGGTTCCGTCGGTTCT	193	NM_001005407	[37]
	R: ATGCCCGTAGCCATCTTCA			
**h*Cacna1i***	F: ATCGGTTATGCTTGGATTGTCA	203	NM_021096	[37]
	R: TGCTCCCGTTGCTTGGTCTC			
**h*GAPDH***	F: CCCATGTTCGTCATGGGTGT	145	NM_002046	[38]
	R: TGGTCATGAGTCCTTCACGATA			
**m*Cacna1g***	F: GGAGCTGGAGCTAGAGATGA	371	NM_009783	[39]
	R: CAGACAAGATGGAGCCTGACT			
**m*Cacna1h***	F: TCTCTGAGCCTCTCACGGAT	300	NM_021415	[39]
	R: GATGTGGCTGACCTCCTCAT			
**m*Cacna1i***	F: CTGGAGACCTGGATGAATGCT	326	NM_001044308	[39]
	R: CAAGAGGGTGCAGTTGACACT			
**m*GAPDH***	F: CATGGCCTTCCGTGTTCCTA	421	NM_008084	[38]
	R: GCGGCACGTCAGATCCA			

^1^ Pairs of primers used to amplify fragments of human (h) and mouse (m) genes encoding T-type calcium channels Cav3.1 (*Cacna1g*), Cav3.2 (*Cacna1h*) and Cav3.3 (*Cacna1i*). Amplification of *GAPDH* encoding glyceraldehyde 3-phosphate dehydrogenase was performed for control of the integrity and amounts of cDNAs used as templates.
